# What Are the Most Prevalent Welfare Issues for Pet Small Mammals?

**DOI:** 10.3390/ani15101423

**Published:** 2025-05-14

**Authors:** Lauren Fenton, Livia Benato, Elisabetta Mancinelli, Nicola J. Rooney

**Affiliations:** 1 Bristol Veterinary School, University of Bristol, Langford BS40 5DU, UK; laurenfenton2002@gmail.com (L.F.); livia.benato@gmail.com (L.B.); 2 Experimental Animal Center (EAC), University of Bern, Murtenstrasse 31, CH-3008 Bern, Switzerland; 3 Royal Veterinary College, Royal College Street, London NW1 0TU, UK; emancinelli@rvc.ac.uk

**Keywords:** animal welfare, rabbits, guinea pigs, hamsters, rats, mice, chinchillas, degus, gerbils, veterinary

## Abstract

The welfare of pet small mammals is an understudied, but growing research topic in the UK and Europe. We surveyed European veterinary experts who each rated severity and prevalence of a large number of issues in the eight most common species. Overall, 46 responses were obtained. We calculated the mean expert’s ratings for severity and prevalence of each issue, which were multiplied together to create an overall impact score. We then ranked issues from most to least impactful for each species. The most impactful welfare issue was perceived to be small housing for all the species apart from the rat where the main concern was respiratory disease. Where comparing veterinary surgeons and veterinary nurses, rating differed significantly on numerous issues. Veterinary nurses generally rated welfare issues as more prevalent than veterinary surgeons. UK respondents also rated numerous issues as more severe and prevalent than non-UK respondents. Better guidance on, and increased availability of, affordable large housing is likely to greatly improve small mammal welfare.

## Simple Summary

The welfare of pet small mammals is an understudied, but growing research topic in the UK and Europe. We used a questionnaire to poll the opinions of experts about the prevalence and severity of multiple welfare concerns, for each of the eight most common species (rabbits, guinea pigs, hamsters, rats, mice, chinchillas, degus, and gerbils). Using their ratings, we derived a welfare impact scale and compared the opinions of different professionals across different countries. There were a large number of issues raised for all species. Small housing was the welfare issue rated as the most impactful for seven of the species, whilst for rats it was respiratory disease. Inappropriate diets also figured prominently for all species. Opinions varied between professionals, for example veterinary nurses rated a variety of welfare issues as more prevalent than did veterinary surgeons. Efforts can now be directed towards addressing the most impactful issues for each species. Many owners are currently not meeting their companion animals’ welfare needs and so improved education and regulation is required, as well as greater availability of larger housing and appropriate diets. By concentrating efforts on these issues, we believe maximum welfare improvement can be achieved.

## 1. Introduction

Keeping companion small mammal pets has increased in popularity around the world [1]. The most common pet small mammal in the UK is the rabbit, and 800,0000 animals are kept in 2% of UK homes [2]. The second most common is the guinea pig, with just over one million kept in 1.8% of UK homes [2]. There are approximately 900,000 hamsters [2] whilst other common UK pet rodents include rats, mice, gerbils, and chinchillas [3] and 0.4% of UK citizens own degus [4]. Although commonly kept, there are relatively few scientific studies on these species’ welfare.

In the UK, owners have a legal requirement under the Animal Welfare Act [5] that states carers must provide for an animals’ five welfare needs: (1) a suitable environment/place to live; (2) a suitable diet; (3) to exhibit normal behaviour patterns; (4) to be housed with or apart from other animals; and (5) to be protected from pain, injury, suffering, and disease [5]. There exists considerable information on how to meet these needs and provide optimal care of cats and dogs (6), and in some countries Codes of Practice exist for rabbits [6,7]. In Europe, the Treaty on the Functioning of the European Union (TFEU) Article 13 states that “the Union and the Member States shall, since animals are sentient beings, pay full regard to the welfare requirements of animals” [8]. Although both pieces of legislation include all vertebrate species, “non-traditional companion animals” are less likely to have their welfare needs met [9]. Small mammals can be owned by anyone over the age of 16, without knowledge of their husbandry, welfare, or biology [10] and vendors do not have to provide information about how to care for them. Consequently, many owners lack knowledge [9]. In addition, social attitudes of not taking small animals to the veterinary clinic [9], and some species being unsuited to the European climate (e.g., temperature, light, humidity, food availability), may all compromise their welfare. Evidence-based information on how best to meet the needs for each of the small mammal species is currently lacking, as is information on the extent to which owners currently meet these needs and hence the most pertinent welfare issues for each small mammal species. There is a growing body of research on rabbits (e.g., [11,12,13,14,15,16,17,18,19]) but relatively little on other small mammal species.

Previous research quantifying the severity of welfare issues in companion animals includes the generic illness severity index for dogs “GISID” [20], which looked at the severity of disorders identified in pedigree dogs. A study by Rioja-Lang et al., [21] ranked and prioritised UK rabbit welfare issues using expert consensus, as did a study by Rooney et al. (described in [14]), which used a survey of 1254 rabbit owners in the UK [15] as well as expert opinions, to prioritise rabbit welfare issues. However, such studies have been restricted to rabbits and to the UK. In this paper, we therefore explore the most important welfare issues in a range of the most common species kept as pets within Europe.

Following a similar methodology to Rooney et al. [14], we surveyed small mammal veterinary experts who each rated the severity and prevalence of a range of welfare issues in each of eight common small mammal species. We used their ratings to derive “importance” scores and thereby constructed a prioritised list of welfare issues for each species. Veterinary care is undertaken by an interprofessional team, with veterinary surgeons and nurses having different experiences [22], and having been shown to have subtlety different opinions as to the severity of issues, for example during pain scoring [22,23]. There are also known differences in legislation and cultural attitudes to welfare between countries. Therefore we explored whether ratings differed between veterinary surgeons and veterinary nurses and between those in the UK and other locations.

## 2. Materials and Methods

### 2.1. Ethical Approval

Ethical approval was granted by the University of Bristol, Health Sciences Student Research Ethics Committee (HSSREC Ref: 16941).

### 2.2. Survey Development

The eight most common small mammal species, as identified in UK survey data ([2,3], rabbits, guinea pigs, Syrian hamsters, rats, mice, chinchillas, degus, and gerbils), were selected for study.

Rooney et al. [14] identified 46 key welfare issues in companion rabbits. Each was described in an objective and quantifiable way. We started from this list for rabbits and extracted all those relevant to each of the other species, conducting a full literature search to identify other species-specific issues. When asking about the issue of small housing, to try to align respondents’ perceptions, we quoted sizes based on median cage size described from surveys of 666 rabbit [13] and 4590 guinea pig owners [24] and minimum sizes advised by the Blue Cross [25], for other species. This resulted in lists between 17 and 33 issues for each species, which were used in our survey.

The survey was divided into twelve sections and included a participant information sheet (Supplementary Materials S1). The second section was demographics: asking information about the participant, e.g., their profession, qualifications/specialty, and the country they currently lived in. The third section, “your experience”, asked how often the participant encountered each of the species. Sections 4–11 asked the participants’ opinions on welfare issues in each of the eight species: rabbit, guinea pig, Syrian hamster, rat, mouse, chinchilla, degu, and gerbil. In Section 4, they were first asked to estimate prevalence: “Please rate approximately what percentage of your country’s rabbit population you think are affected by each of the following issues during their lifetime”; options given were 0%, less than 10%, 10–24%, 25–49%, 50–74%, 75% or more, and 100%. Next, they rated the severity of each issue for rabbits on a scale of 1–5 (1-least severe 5-most severe), i.e., the extent to which they believed the issue would commonly impact upon an animals’ welfare at a given point in time. Sections 5–11 repeated these questions with reference to each of the species in turn. The final section asked the participant to add any comments about the survey. In total, there were 75 questions, with up to 31 welfare issues for each of the species, overall, 440 variables were collected from each participant.

The survey was piloted on five people and minor adjustments to wording were made in light of their feedback. It was uploaded on Jisc™ online surveys and was active from 23 January 2024 to 1 February 24.

### 2.3. Survey Distribution

We targeted experts within the veterinary field with exotic/small mammal knowledge or qualifications.

Emails were sent Secialists of the European College of Zoological Medicine (ECZM) and European College of Animal Welfare and Behavioural Medicine (ECAWBM), both of which operate under the European Board of Veterinary Specialisation (EBVS) and to members of the BVZS (British Veterinary Zoological Society. Snowball sampling was then used by asking participants to pass on the survey to any other exotic specialists they knew.

### 2.4. Analysis

Responses were downloaded into Microsoft Excel, coded, and transferred into IBM SPSS™ Statistics 25 for statistical analysis. Means and standard deviations were calculated for each of the welfare issues in each species for both severity and prevalence. A welfare impact score was derived for each issue for each species by multiplying the mean severity and prevalence and ordering from least to most impactful. The data were not normally distributed so non-parametric tests were used throughout [26]. Since each respondent rated each species, Friedmans tests [27] were used to explore differences in average ratings for the 14 shared welfare issues across the different species. For demographic comparisons, rabbits were chosen as they were the most common small mammals species seen in veterinary practice [28]. The ratings for prevalence and severity of each issue in rabbits were compared between veterinary surgeons and nurses and between participants in and outside the UK, using Mann–Whitney U-tests [29]. Since this is exploratory analysis, we assumed significance at *p* < 0.05.

## 3. Results

### 3.1. Respondents

There was a total of 46 participants. The majority were from the UK (29) whilst the remainder were from seven European countries (14) and two places outside of Europe (2; Canada and Hong Kong; Table 1). Of the 46 participants, 28 were registered veterinary surgeons (VS), and 16 were registered veterinary nurses (RVN) of which 10 had a diploma, 4 a Bachelor of Science (BSc) degree, and 2 a Foundation in Science (FdSc) degree. There was also one student veterinary nurse and a veterinary care assistant (Table 1).

Overall, 14 respondents reported being members of the EBVS (European Board of Veterinary Specialisation). A total of eight different specialisms/qualifications were reported, the most common being ECZM (Table 1). Eleven participants specified having other qualifications/certificates not listed, including Advanced Programme of Veterinary Nursing of Exotic Species, BSc Animal Management, and Certificate in Exotic Veterinary Nursing.

Most of the participants had been informed about the survey via email (26), but five had been referred by a friend/colleague. Overall, 14 of the participants found the survey through social media as the British Veterinary Nursing Association (BVNA) shared the it via Instagram and Facebook, whilst one stated the British Veterinary Zoological Society (BVZS) group (Table 1).

**Figure 1:**
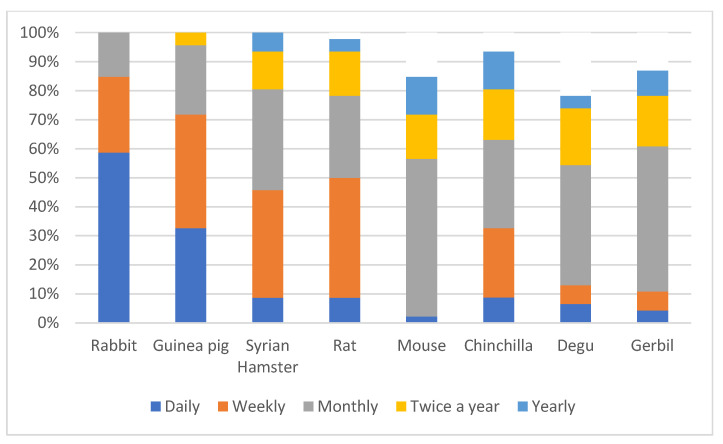
Frequency with which participants reported encountering each of the eight species at work in the order presented in the survey.

Rabbits were the most frequently encountered species with 58.7% of participants seeing them daily whilst at work (Figure 1). Syrian hamsters and rats were most commonly encountered weekly (37% and 41.3%), whilst the most respondents reported encountering mice (54.3%), gerbils (50%), chinchillas (30.4%), and degus (41.3%) monthly (Figure 1).

### 3.2. Most Impactful Welfare Issues

When impact was calculated using mean prevalence and severity scores, the most impactful issue for rabbits was found to be their living space being too small, which had an overall impact of 20.96 (Table 2). This was followed by gut stasis, dental issues, inappropriate diet, and lack of companion rabbit. Similarly, the highest ranked welfare issue was small housing for guinea pigs, hamsters, mice, degus, gerbils, and chinchillas. The rat was the only species where small housing was not the most impactful issue; it came fourth whilst respiratory disease was ranked highest, with an impact score of 23.05 (the highest of all the ratings).

Species comparison

Of the 14 issues compared, 12 prevalences and 10 severities differed significantly amongst species (Table 3 and Table 4). Inappropriate handling, gastrointestinal issues, parasites, and sore hocks/bumblefoot were all rated as most common and most severe in rabbits. Small housing was believed to be most prevalent in hamsters, but most severe in rabbits. Musculoskeletal disorders were considered most prevalent in rabbits but most severe for guinea pigs. Living with an incompatible member of the same species, which causes fights and/or fear, was rated as most prevalent in guinea pigs, but most severe in hamsters. Inappropriate diets and being overweight were both considered most common in hamsters and most severe for rabbits. Inability to display normal behaviours for that species was rated as similarly severe in all species, but least prevalent in mice. Being underweight was considered most prevalent in chinchillas and most severe in rabbits. Dental issues were rated as most prevalent in chinchillas and most severe for guinea pigs. Absence of toys and objects to interact with was considered most common in guinea pigs. Living near predator species, e.g., dogs and cats, did not differ in either its prevalence or severity amongst species.

The overall percentage of the population affected and severity differed significantly amongst species. The prevalence of welfare issues displayed by guinea pigs was rated the highest, whilst degus were rated the lowest. Participants rated the overall severity of the issues highest for rabbits, and lowest for mice (Table 4).

### 3.3. Comparison Between Veterinary Surgeons (VS) and Veterinary Nurses’ (RVN) Opinions

Of 31 welfare issues rated for rabbits, 13 issues that were rated significantly different for prevalence by vets compared to nurses, all of which were rated as more prevalent by nurses. In addition, one issue, living too close to loud noise, was rated as significantly more severe by nurses than by vets (Table 5).

### 3.4. Comparison of Opinions by UK Participants and the Rest of the Population

Of the 31 rabbit welfare issues, there were seven issues that were rated significantly differently for prevalence by participants in the UK compared to those outside of the UK, whilst three were rated differently for severity; all were rated as more prevalent or more severe by UK participants (Table 6).

## 4. Discussion

There were a large number of potential welfare issues identified for each of the species, showing the multiple challenges to meeting their welfare needs as pets. Whilst they have all been kept as pets for many years, knowledge of how best to meet their needs has clearly not fully infiltrated the pet-owning community. There was consensus amongst respondents that some issues are more prevalent and more severe than others, and hence if we are to improve animal welfare, we should target those issues deemed most impactful. Respondents overall rated the prevalence of issues in guinea pigs as the highest, whilst overall severity was rated highest in rabbits. This may reflect true interspecies differences in the occurrence and severity of issues or may reflect differences in familiarity with species, as rabbits and guinea pigs represent the highest case load.

The issue scoring the highest impact, of the 31 considered for rabbits, was small housing. Rooney et al., (described in [14]) prioritised welfare issues for pet rabbits based on stakeholders’ opinions and on a survey of prevalence in the UK [15]. When ranking was based on the severity of issues to a given animal throughout its life, inadequate hutch sizes featured prominently, but when prevalence was also considered solitary living, unpredictable routines, lack of human contact, digging and grazing opportunities were ranked higher. A Delphi survey by Rioja-Lang et al., [21] also described inadequate housing/environment, as the most prevalent welfare issue in UK pet rabbits. Although categorising issues slightly differently, Roelof’s review of the most common welfare issues also identified small housing as well as lack of companions as major issues [30]. Hence our findings reinforce the importance of housing size as an issue for rabbits.

Small housing was also rated as the most impactful issue in other species such as guinea pigs, Syrian hamsters, mice, chinchillas, and degus. It was rated similarly by both veterinary surgeons and nurses and within and outside the UK. This is unsurprising, as housing conditions are generally long lasting and affect an animal’s physiology and behaviour [31]. Previous experimental studies have demonstrated the detrimental effects of restricted housing size on a variety of species. Rabbits in small cages (0.88 m^2^) showed increased inactivity, and were prevented from hopping, stretching, and rearing [32] whilst pairs of rabbits in small hutches (0.73 m^2−^ similar to those commonly on sale for pets), and those with restricted access to exercise areas, showed behavioural and physiological indicators of stress [16]. Fischer et al. [33] found that hamsters in small cages displayed stereotypical wire-gnawing for significantly longer and more frequently than those housed in larger cages. When given the option, mice showed a preference for larger cages [34].

For rats, it is not just two-dimensional cage area that was considered important. Limited vertical space was rated as the third most impactful issue. The UK Animals Scientific Procedures Act 1986 Code of Practice [35] and the standards of the EU directive [36] both state that rats must be kept in a cage with minimum height of 18 cm (about 7.09 in). With no legislation regarding pet cage sizes, many pet cages sold commercially, adhere to the same recommended sizes [37]. However, adult rats can rear up to 30 cm (about 11.81 in), meaning they cannot stand upright in most cages [38]. Vertical space creates new hiding spots, alternative environments, and stimulates motility [38]. Given the opportunity, rats regularly stand upright for exploration and social behaviour [38]. Therefore, limiting cage height likely compromises their welfare [39].

Despite all this knowledge, small housing is still prevalent for all species. The reason is likely twofold, a lack of knowledge of appropriate sizes amongst owners, coupled with a lack of availability of affordable larger housing. Improvement therefore likely requires not just greater owner education, which is isolation is unlikely to greatly impact animal welfare [40], but also regulation of retailers to prevent the sale of inadequate housing. Studies have shown that owners’ choice of how to house their rabbits is influenced by the housing observed in the pet outlet [41] and hence regulation of how animals can be exhibited could also be beneficial. Since housing size was universally rated to be both a prevalent and severe problem, this issue needs to be addressed as a matter of priority.

Unlike other species, for the rat, the highest-ranking issue was respiratory disease. This was the highest impact score of all issues across all species, showing it was believed to be both very severe and highly prevalent. Respiratory disease caused by infectious agents has previously been identified as the most common health problem in rats [42]. A study of 677 rat owners saw 60.4% of the participants report respiratory issues in their pet rat [43]. Respiratory disease has many causes and contributing factors including poor husbandry, stress, cardiovascular disease, exposure to infectious agents, and poor diet [44], and it can significantly impact rat health and welfare. Enhanced owner education about housing and care could therefore help to prevent this important issue.

Inappropriate diet was one of the top five most impactful issues for all the species except for the rat where it featured seventh. It was the second most impactful issue for guinea pigs, hamsters, and gerbils. The complex dietary requirements of these rodents and their need for a constant food source (they naturally eat at approximately 2 h intervals throughout the day [45]) may not be fully understood and may be difficult for owners to maintain. Hence these species often do not receive the correct diet, resulting in health issues. There were numerous other highly rated issues that are also known to be directly related to inappropriate diets. For example, dental disease was in the top five most impactful issues for rabbits, guinea pigs, chinchillas, and degus. The links between dental disease and poor diet due to inadequate fibre intake, excessive sugar, and an imbalance of calcium/phosphorus are well established [46]. In the wild, these species graze for a large proportion of the day. When in captivity, without ad lib grazing and foraging, they can suffer from insufficient fibre intake [47], preventing their teeth from being worn down, which can cause severe malocclusions, inability to eat and potential starvation [48]. Education of potential owners regarding species-specific dietary requirements and sale of high-quality feeds and forage are therefore vital.

Although we aimed to recruit behaviourists (and emailed all European College of Animal Welfare and Behaviour Specialists) as well as veterinary nurses and surgeons, our sample contained no behaviour specialists. However, behavioural issues still featured prominently in the top issues identified. Inability to display normal behaviours was in the top five most impactful issues for hamsters, chinchillas, degus, and gerbils. This is intimately linked to many of the other welfare issues, for example small housing, lack of companions (depending on the species) and lack of environmental enrichment [49]. Lack of companions was also one of the top five most impactful issues for rabbits, chinchillas, and degus. Caviomorphs (degus and chinchillas) are known to more stressed when kept alone [46]. Rabbit owners report higher levels of negative behaviours and more signs of ill health in rabbits kept alone [14], whilst rabbits housed in pairs in rescue centres exhibit less bar biting, higher body temperatures, and recover more quickly from the stress of handling compared to those housed alone [50]. In addition, inappropriate handling was identified as very important for the smaller rodents: hamsters, gerbils, and mice. The inclusion of these behavioural issues in the top ranking, in spite of the respondents all being veterinary nurses and surgeons who are predominantly physical health-oriented professionals, is testimony to the importance of behavioural issues for small mammals.

To compare veterinary surgeon and nurses’ opinions, we analysed their responses for rabbits and found several significant differences. Whilst rating severity, the two groups were quite similar (except loud noises being rated higher by nurses), but their perception of the prevalence of the issues showed greater differences. Nurses reported the percentage of the population affected by 12 of the 31 issues as significantly higher than did veterinary surgeons. Whilst a difference in perceived severity may have been expected based on past research [22], the reasons for these differences in perceived prevalence are less clear. One explanation could be that veterinary surgeons typically receive training focusing predominantly on pathology, diagnosis, and treatment. Hence, they may notice a greater number of these issues. It is also possible that the veterinarians may balance welfare with practical evidence-based or economic considerations as well, potentially resulting in more utilitarian judgements. By contrast, nurses tend to spend more time in direct contact with animals during recovery and routine care, allowing them to observe subtle behavioural changes. This may heighten their sensitivity to welfare concerns and hence they notice more and rate prevalence higher. It is plausible that nurses have a more accurate impression of prevalence than do vets, who have more time limited and focused consultations. However, the accuracy of their respective estimates warrants further investigation. Whilst we only analysed these differences for rabbits, we have no a-priori reason to assume that the results would be different for the other species.

When comparing UK to non-UK veterinary professionals, the former rated ten of the 31 rabbit welfare issues higher for either severity (three issues) or prevalence (seven issues) than did participants from outside the UK. Living near predator species was rated significantly higher for both severity and prevalence. These disparities could reflect differences in perceptions of what is acceptable. For example, the UK Department for Environment Food and Rural Affairs states “Predators and prey must not be kept within sight, sound or smell of each other” [51]. Consequently, many veterinary clinics offer separate areas to avoid predator and prey species coming into contact. The Rabbit Welfare Association and Fund (RWAF) has a rabbit friendly vet directory containing vet practices that have been reviewed and approved by a specialist veterinary adviser to have a high standard of expertise [52]. This may result in UK veterinary professionals being more aware of the potential impact of predators and prey living in close proximity. However, the European Parliament and the Council of the European Union also state that “Species that are incompatible, for example predator and prey, shall not be housed in the same room nor, within sight, smell or sound of each other” [53]. Although scientific evidence examining the effects of habitual contact between predator and prey species may suggest that this could lead to prolonged activation of the hypothalamic–pituitary–adrenal (HPA) axis in prey species, resulting in chronic stress, behavioural changes, and impaired welfare in prey species, the situation may be different particularly in the context of captivity [54]. Partial habituation over time (for example where different species are inappropriately housed together but get used to each other over time) may result in physiological stress markers decreasing with repeated non-threatening exposure [55]. However, in many species, persistent predator cues (e.g., sight, sound, or scent) maintain high stress levels, indicating ongoing perceived threat [56]. The higher ratings for prevalence of numerous issues reported by UK veterinary professionals may reflect the issues being more common in the UK compared to other European countries or could be due to UK respondents having different thresholds of what is acceptable, being more informed and hence more vigilant regarding some of the welfare issues. The reason for the differences requires further study, but it does highlight differences between nations in occurrence and/or perceptions of veterinary professionals.

A limitation of this study is the relatively small number of 46 veterinary professional respondents. However, veterinary surgeons are notoriously time poor and hence recruiting them to fill in a relatively long (20 min) questionnaire was challenging. It is likely that those completing it were especially passionate about small mammal welfare, so their responses may not be representative of the general veterinary community. However, we were keen to recruit the most expert small animal practitioners and to ask about all species to allow interspecies comparison. Future work should aim to confirm these findings using clinical data for comparison to opinions on prevalence voiced here. It should concentrate on a smaller number of issues as prioritised here, to facilitate a large scale but targeted survey that reaches a wider veterinary population. It should also seek different ways to target multiple European countries. Despite the European Board of Veterinary Specialists (EBVS) backing our survey, it reached only a small number of participants. Increased sample sizes will allow meaningful inter-country comparisons.

## 5. Conclusions

Although pet small mammals are subject to many welfare issues as suggested by this study, prioritisation of these issues can help to determine those that most impact upon animal welfare. Our study effectively created prioritised welfare lists for each of eight pet small mammal species and compared the opinions of different veterinary professionals, and UK vs. non-UK. Whilst studies on rabbit welfare have increased recently, we have included the often-neglected other pet small mammal species.

The most impactful issues reported in this study are commonly seen by veterinary professionals in practice, which suggests that many owners do not have the knowledge or resources to care for their pets correctly. Veterinary professionals should continue to raise awareness regarding the most important issues and educational resources should prioritise improving owner understanding of these commonly seen welfare issues. The finding that so many of the welfare needs are often unmet, raises the question as to why owners procure animals without seeking out evidence-based information on how to meet their welfare needs. There is currently no requirement for information and advice to be given prior to an outlet selling or an owner obtaining a pet small mammal.

We suggest that when resources are limited, veterinary organisations and charities should prioritise resources for the welfare issues deemed to be most impactful for each species. This has been achieved through a coordinated Rabbit Welfare Strategy in the UK, [14], which prioristises the development of statutory codes of practice for owners and aims to set up systems for dissemination of consistent evidence-based information to potential owners, at point of sale. A similar approach for other species and in other countries may be beneficial.

The single most impactful welfare issue was found to be small housing for all the species apart from the rat, where the main concern was respiratory disease. Housing should be a prioritised area for improvement across all species and across Europe. One way to have rapid impact would be to regulate pet stores that sell enclosures to ensure that all cages sold to small mammal pet owners meet a minimum cage size (perhaps based on the size of the average adult pet).

## Figures and Tables

**Table 1:** Characteristics of participants completing the survey.

Characteristic	Answer Given	Number of Respondents (n = 46 Total)	Percentage of Respondents (%)
Country	UK	29	63
	Canada	1	2.2
	Hong Kong	2	4.3
	Italy	4	8.7
	Denmark	3	6.5
	Finland	2	4.3
	Czech Republic	2	4.3
	Germany	1	2.2
	Netherlands	1	2.2
	France	1	2.2
Profession	Veterinary surgeon (VS)	28	60
	Veterinary nurse (RVN)	16	34.8
	Student veterinary nurse (SVN)	1	2.2
	Veterinary care assistant (VCA)	1	2.2
Board memebership	Part of the EBVS (European Board of Veterinary Specialisation)	14	30.4
Recognised specialist qualification/certificate in any discipline	ECZM (European College of Zoological Medicine) specialists	12	26.1
	ECAWBM (European College of Animal Welfare and Behavioural Medicine) specialist	0	0
	EBVS (European Board of Veterinary Specialisation) specialists	5	10.9
	RCVS (Royal College of Veterinary Surgeons) specialist	6	13
	ACZM (American College of Zoological Medicine) specialist	1	2.2
	ABVP (American Board of Veterinary Practitioners)	1	2.2
	RCVS (Royal College of Veterinary Surgeons) Certificate	4	8.7
	AVP (Advanced *Veterinary* Practice) Certificate	17	8.7
	Other	11	23.9
	None	17	37
Method of finding out about the survey	Email	26	56.5
	Friend	5	10.9
	Social media	14	30.4
	Other	1	2.2

**Table 2:** Top five most impactful welfare issues as rated by respondents for each of the eight pet small mammal species. The full list of all issue can be found in Supplementary Materials Ranking highest to lowest.

	Welfare Issue	Mean Rating for Severity of the Issue (±sd: Scored 1–5)	Means Rating for Proportion of Population Affected (±sd: Scored 1–5)	Impact (Maximum 25)
**Rabbits**				
1	Living space too small (e.g., less than 1 m × 0.6 m) per rabbit	4.46 ± 0.91	4.70 ± 1.31	20.96
2	Gut stasis	4.41 ± 0.91	4.39 ± 1.36	19.36
3	Dental issues	4.35 ± 0.92	4.41 ± 1.09	19.18
4	Inappropriate diet (e.g., lack of hay/grass)	4.46 ± 0.94	4.20 ± 1.19	18.73
5	Lack of companion rabbit	4.09 ± 1.09	4.52 ± 1.03	18.49
**Guinea pigs**				
1	Living space too small (e.g., less than 0.53 m^2^ per guinea pig)	4.23 ± 1.01	4.73 ± 1.45	20.01
2	Inappropriate diet (e.g., lack of hay/grass)	4.42 ± 0.85	4.52 ± 1.30	19.98
3	Dental issues	4.36 ± 0.92	4.39 + 0.97	19.14
4	Urinary system disorders	4.20 ± 0.98	4.41 ± 1.35	18.52
5	Being overweight	3.80 ± 1.00	4.77 ± 1.12	18.13
**Syrian hamster**				
1	Small housing e.g., under 100 cm × 50 cm floor (5000 cm^2^)	4.16 ± 1.20	4.82 ± 1.56	20.05
2	Inappropriate diet	4.07 ± 0.9	4.59 ± 1.45	18.68
3	Inappropriate handling	4.05 ± 1.13	4.59 ± 1.56	18.59
4	Inability to display normal behaviours (e.g., burrowing, foraging, gnawing)	4.11 ± 1.15	4.18 ± 1.50	17.18
5	Being overweight	3.66 ± 1.12	4.64 ± 1.28	16.98
**Rat**				
1	Respiratory disease	4.39 ± 0.84	5.25 ± 1.04	23.05
2	Tumours/masses	3.82 ± 0.90	5.09 ± 1.12	19.44
3	Limited vertical space	4.25 ± 0.84	4.34 ± 1.29	18.45
4	Small housing e.g., under 90 cm length × 60 cm width per two rats (5400 cm^2^)	4.30 ± 0.95	4.27 ± 1.45	18.36
5	Being overweight	3.68 ± 1.05	4.61 ± 1.08	16.96
**Mouse**				
1	Small housing e.g., under 80 cm × 50 cm floor space per two mice (4000 cm^2^)	4.07 ± 1.30	4.43 ± 1.85	18.03
2	Respiratory disease	4.09 ± 1.20	3.98 ± 1.32	16.28
3	Tumours/masses	3.61 ± 1.17	4.27 ± 1.47	15.41
4	Inappropriate handling	3.57 ± 1.23	4.11 ± 1.56	14.67
5	Inappropriate diet	3.43 ± 1.13	4.07 ± 1.47	13.96
**Chinchilla**				
1	Small housing e.g., under 90 cm length × 60 cm width per 2/3 chinchillas (5400 cm^2^)	4.38 ± 1.03	4.56 ± 1.47	19.97
2	Dental issues	4.13 ± 1.06	4.62 ± 1.27	19.08
3	Lack of chinchilla companion	4.04 ± 1.19	4.36 ± 1.46	17.61
4	Inappropriate diet	3.93 ± 1.12	4.27 ± 1.36	16.78
5	Inability to display normal behaviours (e.g., climbing, foraging, gnawing)	4.09 ± 1.04	4.02 ± 1.37	16.44
**Degu**				
1	Small housing e.g., under 90 cm length × 60 cm width per two degus (5400 cm^2^)	4.07 ± 1.29	4.18 ± 1.79	17.01
2	Dental issues	3.84 ± 1.21	4.13 ± 1.53	15.86
3	Inappropriate diet	3.73 ± 1.23	4.18 ± 1.50	15.59
34	Inability to display normal behaviours (e.g., climbing, foraging, digging, gnawing)	3.80 ± 1.25	3.76 ± 1.57	14.29
5	Lack of degu companion	3.84 ± 1.22	3.69 ± 1.61	14.17
**Gerbil**				
1	Small housing e.g., under 100 cm long, by 40 cm wide per 2–4 gerbils (4000 cm^2^)	4.14 ± 1.21	4.27 ± 1.81	17.68
2	Inappropriate diet	3.73 ± 1.13	4.23 ± 1.63	15.78
3	Inappropriate handling	3.66 ± 1.16	4.27 ± 1.61	15.63
4	Living with an incompatible gerbil, which causes fights and/or fear	4.05 ± 1.17	3.66 ± 1.54	14.82
5	Inability to display normal behaviours	3.77 ± 1.29	3.89 ± 1.79	14.67

**Table 3:** Comparisons of the ratings for prevalence and severity of the 14 common welfare issues amongst all eight small mammal species (rabbits, guinea pigs, hamsters, rats, mice, chinchillas, degus, and gerbil).

Welfare Issue	Prevalence		Severity	
	Friedman Test	*p*-Value	Friedman Test	*p*-Value
Small housing	14.65	0.041	17.19	<0.016
Musculoskeletal disorders	40.98	0.001	28.17	<0.001
Living with an incompatible member of the same species, which causes fights and/or fear	31.45	0.001	27.30	<0.001
Inappropriate diet	17.44	0.015	73.70	<0.001
Inability to display normal behaviours for that species	13.73	0056	18.60	<0.010
Inappropriate handling	39.65	0.001	24.17	<0.001
Being underweight	17.62	0.014	38.98	<0.001
Being overweight	66.67	0.001	56.46	<0.001
Dental issues	100.35	0.001	58.25	<0.001
Gastrointestinal issues	87.48	0.001	92.80	<0.001
Parasites	29.43	0.001	33.99	<0.001
Sore hocks/bumblefoot	70.58	0.001	39.13	<0.001
Absence of toys and objects to interact with	26.14	0.001	5.82	0.562
Living near predator species e.g., dogs and cats	8.82	0.266	8.13	0.321
Mean for all issues	34.11	<0.001	61.91	<0.001

**Table 4:** Participants’ ratings (median, 25th and 75th percentiles) for the percentage of the population affected and the severity of the 12 welfare issues shared by the eight species, which were seen to differ significantly between species (n = 46).

Issue	Median (25th and 75th Percentiles)							
	Rabbit	Guinea Pig	Syrian Hamster	Rat	Mouse	Chinchilla	Degu	Gerbil
**Percentage of the Population Affected**								
Small housing	5 (4, 6)	5 (4, 6)	5 (4, 6)	4 (4, 4)	5 (3, 6)	5 (3, 6)	5 (3, 6)	5 (3, 6)
Musculoskeletal disorders	4 (3, 5)	4 (3, 5)	3 (2, 5)	5 (3.5, 5.5)	3 (2, 4)	3 (2.25, 4)	4 (3, 5)	4 (3, 5)
Living with an incompatible member of the same species, which causes fights and/or fear	4 (4, 5)	4 (4, 4.5)	3 (2, 5)	3 (2, 4)	4 (2.5, 5)	3 (2, 4)	3 (2., 5)	4 (2, 4)
Inappropriate diet	4 (3, 5)	4 (3, 4)	3(2, 4)	3 (3, 4.5)	4(3, 5)	4(3, 5)	4 (3, 5)	4 (3, 6)
Inability to display normal behaviours	4 (3, 5)	4 (3, 5)	4 (3, 6)	4 (3, 5)	3 (2, 4)	4 (3, 5)	4 (2, 5)	4 (2, 5.25)
Being underweight	2 (2, 3)	2 (2, 3)	3 (2, 3.5)	3 (4, 5)	2 (2, 3)	3 (2, 4)	3 (2, 4)	3 (2, 4)
Being overweight	5 (4, 5)	5 (4, 6)	5 (4, 6)	3 (2, 3)	3 (2, 5)	4 (2.25, 4.75)	4 (2, 5)	4 (3, 5)
Dental issues	5 (3, 5)	4 (4, 5)	3 (2, 5)	5 (4, 5.5)	3 (2, 4)	5 (4, 5.75)	4 (3, 5)	4 (3, 4.25)
Gastrointestinal issues	4 (3, 6)	4 (3, 5)	3 (2, 4)	3 (2, 4)	3(2, 4)	4 (3, 5)	3 (2, 4)	3 (2, 4)
Parasites	3 (2, 3)	3 (2, 3)	3 (2, 3)	3 (2, 4)	3 (2, 4)	2 (2, 3)	2 (2, 3)	2 (2, 3)
Sore hocks/bumblefoot	4 (2, 5)	4 (3, 5)	4 (3, 4)	4 (3, 5)	3 (2, 3)	3 (2, 4)	2 (2, 4)	2 (2, 4)
Absence of toys and objects to interact with	4 (3, 5)	4 (3, 5)	3 (2, 5)	3 (2, 3.5)	4 (2, 5)	3 (2.25, 4)	4 (2, 4)	3.5 (2, 5)
Overall mean for all 14 issues	3.9 (3.3, 4.5)	3.9 (3.4, 4.5)	3.8 (3.1, 4.4)	3.6 (2.9, 4.2)	3.6 (2.8, 4.1)	3.8 (3.0, 4.3)	4.4 (3.7, 4.8)	4.2 (3.8, 4.9)
**Severity**								
Small housing	5 (4, 5)	4 (4, 5)	5 (4, 5)	3 (2, 4)	5 (4, 5)	5 (4, 5)	5(4, 5)	5 (4, 5)
Musculoskeletal disorders	4 (3, 4)	4 (3, 5)	4 (3, 4)	5 (4, 5)	3 (2, 4)	3 (3, 4)	3 (2, 4)	3 (2, 4)
Living with an incompatible member of the same species, which causes fights and/or fear	4 (3, 5)	4 (3.5, 5)	5 (4, 5)	3 (3, 4)	4 (3.5, 5)	4 (3.25, 5)	4 (4, 5)	4 (4, 5)
Inappropriate diet	5 (4, 5)	5 (4, 5)	4 (4, 5)	4 (3, 5)	4 (3, 4)	4 (3, 5)	4 (3, 5)	4 (3, 5)
Inability to display normal behaviours for that species	4 (3, 5)	4 (3.5, 5)	5 (4, 5)	4 (3, 4)	4 (3, 4)	4 (4, 5)	4 (3, 5)	4 (3, 5)
Inappropriate handling	4 (3, 5)	4 (3, 4.5)	4 (4, 5)	4 (4, 5)	4 (3, 5)	4 (3, 5)	4 (3, 5)	4 (3, 5)
Being underweight	3 (2, 4)	3 (2, 4)	3 (2, 4)	4 (3, 4.5)	3 (2, 3)	3 (2, 4)	3 (2, 3)	3 (2, 4)
Being overweight	4 (3.5, 5)	4 (3, 4.5)	4 (3, 5)	3 (2, 4)	3 (3, 4)	3 (3, 4)	3 (2, 4)	3 (2, 4.25)
Dental issues	5 (4, 5)	4 (3, 4.5)	4 (4, 5)	4 (3, 4)	4 (3, 4)	5 (3.25, 5)	4 (3, 5)	4 (3, 5)
Gastrointestinal issues	5 (4, 5)	4 (4, 5)	4 (3, 5)	4 (3, 5)	3 (3, 4)	4 (3, 4)	4 (3, 4)	3 (3, 4)
Parasites	3 (2, 4)	3 (2, 4)	3 (3, 4)	3 (3, 4)	3 (2, 3)	3 (2, 3.75)	3 (2, 3)	3 (2, 3)
Sore hocks/bumblefoot	4 (3, 4)	4 (4, 5)	4 (3, 4)	3 (2, 4)	3 (2, 4)	4 (3, 4)	3 (2, 4)	3 (2, 4)
Absence of toys and objects to interact with	3 (3, 4)	3 (2.5, 4)	3 (2, 3.5)	3 (2, 4)	3 (3, 4)	3 (3, 4)	3 (2, 4)	3 (2.75, 4)
Living near predator species	3 (3, 4)	3 (3, 4)	3 (2.5, 4)	4 (3, 4)	3 (2, 4)	3.5 (3, 4)	3 (2, 4)	3 (2.75, 4)
Overall mean- of all 14 issues	3.9 (3, 4, 4.3)	4.0 (3, 3, 4.2)	3.8 (3.4, 4.2)	3.6 (3.4, 4.0)	3.5 (3.0, 3.9)	3.8 (3.4, 4.1)	3.6 (2.9, 4, 0)	3.6 (3.1, 4.0)

**Table 5:** Rabbit welfare issues differing significantly between veterinary surgeons (n = 28) and veterinary nurses (n = 16) when rated for prevalence (proportion of population affected) or severity.

	Median (25th and 75th Percentiles)			
Welfare Issues in Rabbits	VS (n = 28)	RVN (n = 16)	Mann–Whitney U-Value	*p*-Value
**Proportion of population affected**				
Living with a guinea pig, which causes fights and/or fear	2 (2, 3)	3 (2, 4)	311.5	0.021
Lack of vaccinations (myxomatosis/viral haemorrhagic disease)	3 (2.75, 4)	5 (4, 5)	338	0.004
Lack of grooming by owner (matted fur)	3 (3, 5)	5 (3.25, 5.75)	302	0.024
Lack of nail clipping (overly long nails)	3.5 (3, 5)	5 (3.25, 5)	310	0.030
Inappropriate handling	3.5 (2.75, 4.25)	5 (3.25, 5)	320.5	0.016
Living close to loud noises (e.g., fireworks)	3 (2, 4)	4 (3, 5)	306	0.039
Myiasis (flystrike)	2 (2, 3)	3 (2.25, 4)	327	0.008
Bite injuries	3 (2, 4)	3 (2.25, 4)	356.5	0.001
Absence of an exercise area apart from traditional hutch or cage (e.g., run)	3 (3, 4.25)	4 (4, 5.75)	313	0.025
Absence of toys and objects to interact with	3 (2.75, 4)	4 (3.25, 5.75)	318.5	0.018
Living near predator species e.g., dogs and cats	3 (3, 4.25)	5 (4, 5)	332	0.007
**Severity**				
Living close to loud noises (e.g., fireworks)	3 (2, 4)	4 (3, 4)	307	0.034

**Table 6:** Rabbit welfare issues seen to differ significantly between by participants from the UK as compared to those outside of the UK (n = 46), for prevalence and severity.

	Median (25th and 75th Percentiles)			
Welfare Issue	UK (n = 29)	Outside of UK (n = 17)	Mann–Whitney U-Value	*p*-Value
**Prevalence**				
Living with an incompatible rabbit, which causes fights and/or fear	2 (2, 3)	3 (3, 4)	343	0.022
Inappropriate handling	3 (2, 4)	4 (3, 5)	354	0.012
Living close to loud noises (e.g., fireworks)	2 (2, 4)	4 (3, 4)	355.5	0.010
Myiasis (flystrike)	2 (2, 3)	3 (2, 3)	341.5	0.022
Bite injuries	2 (2, 2.75)	3 (2, 4)	351	0.010
Absence of toys and objects to interact with	3 (2, 3)	4 (3, 5)	382	0.002
Living near predator species e.g., dogs and cats	3 (2.25, 3.75)	5 (3, 5)	384	0.001
**Severity**				
Musculoskeletal disorders	3 (2.25, 4)	4 (3,5)	333.5	0.039
Living close to loud noises (e.g., fireworks)	3 (2,3.75)	3 (3,4)	341	0.024
Living near predator species e.g., dogs and cats	3 (2,3)	4 (3,4)	384	0.001

## Data Availability

The original data presented in the study are openly available in University of Bristol.

## Supplementary Materials

The following supporting information can be downloaded at: https://www.mdpi.com/article/10.3390/ani15101423/s1, Supplementary Materials S1: Survey.
