# Supplementary material for: What Are the Most Prevalent Welfare Issues for Pet Small Mammals?
Source: Animals (Basel). 2025 May 14;15(10):1423. doi: 10.3390/ani15101423 (PMC12108152; doi:10.3390/ani15101423)
Supplement: Supplementary file 1 [file animals-15-01423-s001.zip › animals-3537479-supplementary.pdf]

<u>Survey</u>

<u>Demographics:</u>

1. Please indicate whether you are a:
    - Registered veterinary nurse with a veterinary nursing diploma (RVN)
    - Registered veterinary nurse with a BSc degree (RVN)
    - Registered veterinary nurse with a FdSc degree (RVN)
    - Registered veterinary surgeon (VS)
    - Certified companion animal behaviourist
    - Other

2. If other please specify

3. Are you part of the EBVS? (European Board of Veterinary Specialisation)
    - Yes
    - No

4. Do you have a recognized specialist qualification/certificate in any discipline? (e.g. RCVS, European, American, BSAVA, other)?
    - ECZM Specialist
    - ECAWBM Specialist
    - EBVS Specialist
    - RCVS Specialist
    - ACZM Specialist
    - ABVP
    - RCVS Certificate
    - CertAVP
    - Other (please specify)
    - None

5. If yes, please state your specialty, or other qualifications here.

6. How did you hear about the survey?

Was sent an email to participate.

- Friend/colleague
- Social media
- Other

7. If other, please specify.

8. What country do you currently live in?

<u>Your experience:</u>

9. Approximately how often do you encounter each species in your line of work within a given year?

Rabbit:

- Daily
- Weekly
- Monthly
- Twice a year
- Yearly
- Never

Guinea pig:

- Daily
- Weekly

- Monthly
- Twice a year
- Yearly
- Never

Syrian hamster:
- Daily
- Weekly
- Monthly
- Twice a year
- Yearly
- Never

Rat:
- Daily
- Weekly
- Monthly
- Twice a year
- Yearly
- Never

Mouse:
- Daily
- Weekly
- Monthly
- Twice a year
- Yearly
- Never

Chinchilla:
- Daily
- Weekly
- Monthly
- Twice a year
- Yearly
- Never

Degu:
- Daily
- Weekly
- Monthly
- Twice a year
- Yearly
- Never

Gerbil:
- Daily
- Weekly
- Monthly
- Twice a year
- Yearly

- Never

10. Is there any legislation in your country applicable to owners keeping these species? If so, please list below.

<u>Rabbit Welfare:</u>

11. Please rate approximately what percentage of your country's rabbit population you think are affected by each of the following issues during their lifetime.

	0%	Less than 10%	10-24%	25-49%	50-74%	75% or more	100%
Living space too small (e.g., less than 1m x 0.6m) per rabbit							
Living space of inadequate height for rabbit to stand on hind limbs (e.g., less than 0.5m high)							
Lack of places to hide in enclosure							
Musculoskeletal disorders							
Lack of companion rabbit							
Living with an incompatible rabbit which causes fights and/or fear							
Living with a guinea pig which causes fights and/or fear							
Lack of vaccinations (Myxomatosis/Viral haemorrhagic disease)							
Inappropriate diet (e.g., lack of hay/grass)							
Inability to display normal behaviors (e.g., digging, grazing, chinning, binkying)							
Lack of grooming by owner (matted fur)							

Lack of nail clipping (overly long nails)							
Inappropriate handling							
Being underweight							
Being overweight							
Living close to loud noises (e.g., fireworks)							
Dental issues							
Myiasis (flystrike)							
Gut stasis							
Parasites							
Neurological problems							
Sore hocks							
Skin disorders (dermatitis, alopecia, skin lesions)							
Ocular diseases							
Bite injuries							
Encephalitozoon cuniculi infection							
Absence of an exercise area apart from traditional hutch or cage (e.g., run for grazing)							
Absence of toys and objects to interact with							
Living near predator species e.g., dogs and cats							
No opportunity to dig or graze							
Giving birth to more than one litter a year							

12. Please rate the severity of each of the issues on a scale of 1-5 (1-least severe 5-most severe) i.e. the extent to which you believe each condition would commonly impact upon a single rabbit's welfare at a given point in time.

	1	2	3	4	5
Living space too small (e.g., less than 1m x 0.6m) per rabbit					

Living space of inadequate height for rabbit to stand on hind limbs (e.g., less than 0.5m high)					
Lack of places to hide in enclosure					
Musculoskeletal disorders					
Lack of companion rabbit					
Living with an incompatible rabbit which causes fights and/or fear					
Living with a guinea pig which causes fights and/or fear					
Lack of vaccinations (Myxomatosis/Viral haemorrhagic disease)					
Inappropriate diet (e.g., lack of hay/grass)					
Inability to display normal behaviors (e.g., digging, grazing, chinning, binkying)					
Lack of grooming by owner (matted fur)					
Lack of nail clipping (overly long nails)					
Inappropriate handling					
Being underweight					
Being overweight					
Living close to loud noises (e.g., fireworks)					
Dental issues					
Myiasis (flystrike)					
Gut stasis					
Parasites					

Neurological problems					
Sore hocks					
Skin disorders (dermatitis, alopecia, skin lesions)					
Ocular diseases					
Bite injuries					
Encephalitozoon cuniculi infection					
Absence of an exercise area apart from traditional hutch or cage (e.g., run for grazing)					
Absence of toys and objects to interact with					
Living near predator species e.g., dogs and cats					
No opportunity to dig or graze					
Giving birth to more than one litter a year					

13. Please add any additional welfare issues not included above that you think commonly affect rabbit welfare.
Issue 1:
14. Please rate approximately what percentage of the rabbit population you think are affected by the issue you listed above during their lifetime. {{Q13}}

- 0%
- Less than 10%
- 10-24%
- 25-49%
- 50-74%
- 75% or more
- 100%

15. Please rate the severity of the issue on a scale of 1-5 (1-least severe 5 most severe) i.e. the extent to which you believe the condition would commonly impact upon a single rabbit's welfare at a given point in time. {{Q13}}

- 1
- 2
- 3
- 4
- 5

16. Please add any additional welfare issues not included above that you think commonly affect rabbit welfare.
Issue 2:
17. Please rate approximately what percentage of the rabbit population you think are affected by the issue you listed above during their lifetime. {{Q16}}

- 0%
- Less than 10%
- 10-24%
- 25-49%
- 50-74%
- 75% or more
- 100%

18. Please rate the severity of the issue on a scale of 1-5 (1-least severe 5 most severe) i.e. the extent to which you believe the condition would commonly impact upon a single rabbit's welfare at a given point in time. {{Q16}}

- 1
- 2
- 3
- 4
- 5

<u>Guinea Pig Welfare:</u>
19. Please rate approximately what percentage of your country's guinea pig population you think are affected by each of the following issues during their lifetime.

	0%	Less than 10%	10-24%	25-49%	50-74%	75% or more	100%
Living space too small (e.g. less than 0.53m2 per guinea pig).							
Lack of places to hide in enclosure.							
Living with an incompatible guinea pig which causes fights and/or fear.							
Living with a rabbit which causes fights and/or fear.							
Lack of companion guinea pig.							
Inappropriate diet (e.g., lack of hay/grass).							
Scurvy due to lack of Vitamin C.							
Musculoskeletal disorders.							

Abscesses.							
Inability to display normal behaviours (e.g., popcorning, gnawing, grazing).							
Lack of grooming by owner (matted fur).							
Lack of nail clipping (overly long nails).							
Inappropriate handling.							
Being underweight.							
Being overweight.							
Loud noises (e.g., fireworks).							
Disease of the genital system.							
Dental issues.							
Myiasis (flystrike).							
Gastrointestinal disease.							
Parasites.							
Respiratory disease.							
Bumblefoot.							
Ocular diseases.							
Masses/tumours.							
Urinary system disorders.							
Absence of an exercise area apart from traditional hutch or cage (e.g., run for grazing).							
Absence of toys and objects to interact with.							
Living near predator species e.g., dogs and cats.							
No opportunity to graze.							
Skin disease.							

20. Please rate the severity of each of the issues on a scale of 1-5 (1-least severe 5-most severe) i.e. the extent to which you believe each condition would commonly impact upon a single guinea pig's welfare at a given point in time.

	1	2	3	4	5

Living space too small (e.g. less than 0.53m2 per guinea pig).					
Lack of places to hide in enclosure.					
Living with an incompatible guinea pig which causes fights and/or fear.					
Living with a rabbit which causes fights and/or fear.					
Lack of companion guinea pig.					
Inappropriate diet (e.g., lack of hay/grass).					
Scurvy due to lack of Vitamin C.					
Musculoskeletal disorders.					
Abscesses.					
Inability to display normal behaviours (e.g., popcorning, gnawing, grazing).					
Lack of grooming by owner (matted fur).					
Lack of nail clipping (overly long nails).					
Inappropriate handling.					
Being underweight.					
Being overweight.					
Loud noises (e.g., fireworks).					
Disease of the genital system.					
Dental issues.					
Myiasis (flystrike).					
Gastrointestinal disease.					
Parasites.					
Respiratory disease.					
Bumblefoot.					

Ocular diseases.					
Masses/tumours.					
Urinary system disorders.					
Absence of an exercise area apart from traditional hutch or cage (e.g., run for grazing).					
Absence of toys and objects to interact with.					
Living near predator species e.g., dogs and cats.					
No opportunity to graze.					
Skin disease.					

21. Please add any additional welfare issues not included above that you think commonly affect guinea pig welfare.
Issue 1:
22. Please rate approximately what percentage of the guinea pig population you think are affected by the issue you listed above during their lifetime. {{Q21}}

- 0%
- Less than 10%
- 10-24%
- 25-49%
- 50-74%
- 75% or more
- 100%

23. Please rate the severity of the issue on a scale of 1-5 (1-least severe 5 most severe) i.e. the extent to which you believe the condition would commonly impact upon a single guinea pig's welfare at a given point in time. {{Q21}}

- 1
- 2
- 3
- 4
- 5

Please add any additional welfare issues not included above that you think commonly affect guinea pig welfare.
Issue 2:
25. Please rate approximately what percentage of the guinea pig population you think are affected by the issue you listed above during their lifetime. {{Q24}}

- 0%
- Less than 10%
- 10-24%
- 25-49%

- 50-74%
- 75% or more
- 100%

26. Please rate the severity of the issue on a scale of 1-5 (1-least severe 5 most severe) i.e. the extent to which you believe the condition would commonly impact upon a single guinea pig's welfare at a given point in time. {{Q24}}

- 1
- 2
- 3
- 4
- 5

Syrian Hamster Welfare:

27. Please rate approximately what percentage of your country's Syrian hamster population you think are affected by each of the following issues during their lifetime.

	0%	Less than 10%	10-24%	25-49%	50-74%	75% or more	100%
Small housing e.g., under 100cm x 50cm floor (5000cm2)							
Housing with another hamster causing fights							
Inability to display normal behaviours (e.g., burrowing, foraging, gnawing)							
Musculoskeletal disorders							
Wet tail (diarrhea)							
Inappropriate diet							
Inappropriate handling							
Being underweight							
Being overweight							
Dental issues							
Gastrointestinal disease							
Parasites							
Respiratory disease							
Bumblefoot							

Lodged food (in cheek pouches)							
Traumatic injury							
Overgrown nails							
Ocular diseases							
Skin diseases							
Masses/tumors							
Absence of toys and objects to interact with							
Living near predator species e.g., dogs and cats							
Inappropriately sized wheel							

28. Please rate the severity of each of the issues on a scale of 1-5 (1-least severe 5 most severe) i.e. the extent to which you believe each condition would commonly impact upon a single Syrian hamster's welfare at a given point in time.

	1	2	3	4	5
Small housing e.g., under 100cm x 50cm floor (5000cm2)					
Housing with another hamster causing fights					
Inability to display normal behaviours (e.g., burrowing, foraging, gnawing)					
Musculoskeletal disorders					
Wet tail (diarrhea)					
Inappropriate diet					
Inappropriate handling					
Being underweight					
Being overweight					
Dental issues					

Gastrointestinal disease					
Parasites					
Respiratory disease					
Bumblefoot					
Lodged food (in cheek pouches)					
Traumatic injury					
Overgrown nails					
Ocular diseases					
Skin diseases					
Masses/tumors					
Absence of toys and objects to interact with					
Living near predator species e.g., dogs and cats					
Inappropriately sized wheel					

29. Please add any additional welfare issues not included above that you think commonly affect Syrian hamster welfare.
Issue 1:
30. Please rate approximately what percentage of the Syrian hamster population you think are affected by the issue you listed above during their lifetime. {{Q29}}

- 0%
- Less than 10%
- 10-24%
- 25-49%
- 50-74%
- 75% or more
- 100%

31. Please rate the severity of the issue on a scale of 1-5 (1-least severe 5-most severe) i.e. the extent to which you believe the condition would commonly impact upon a single Syrian hamster's welfare at a given point in time. {{Q29}}

- 1
- 2
- 3
- 4
- 5

32. Please add any additional welfare issues not included above that you think commonly affect Syrian hamster welfare.
Issue 2:

33. Please rate approximately what percentage of the Syrian hamster population you think are affected by the issue you listed above during their lifetime. {{Q32}}

- 0%
- Less than 10%
- 10-24%
- 25-49%
- 50-74%
- 75% or more
- 100%

34. Please rate the severity of the issue on a scale of 1-5 (1-least severe 5 most severe) i.e. the extent to which you believe the condition would commonly impact upon a single Syrian hamster's welfare at a given point in time. {{Q32}}

- 1
- 2
- 3
- 4
- 5

<u>Rat Welfare:</u>
35. Please rate approximately what percentage of your country's rat population you think are affected by each of the following issues during their lifetime.

	0%	Less than 10%	10-24%	25-49%	50-74%	75% or more	100%
Small housing e.g., under 90cm length x 60cm width per 2 rats (5400cm2)							
Limited vertical space							
Lack of rat companion							
Living with an incompatible rat which causes fights and/or fear							
Inability to display normal behaviours (e.g., climbing, foraging, gnawing)							
Musculoskeletal disorders							
Tumours/masses							
Inappropriate diet							
Inappropriate handling							

Being underweight							
Being overweight							
Dental issues							
Gastrointestinal disease							
Parasites							
Respiratory disease							
Bumblefoot							
Absence of toys and objects to interact with							
Living near predator species e.g., dogs and cats							

36. Please rate the severity of each of the issues on a scale of 1-5 (1-least severe 5-most severe) i.e. the extent to which you believe each condition would commonly impact upon a single rat's welfare at a given point in time.

	1	2	3	4	5
Small housing e.g., under 90cm length x 60cm width per 2 rats (5400cm2)					
Limited vertical space					
Lack of rat companion					
Living with an incompatible rat which causes fights and/or fear					
Inability to display normal behaviours (e.g., climbing, foraging, gnawing)					
Musculoskeletal disorders					
Tumours/masses					
Inappropriate diet					
Inappropriate handling					

Being underweight					
Being overweight					
Dental issues					
Gastrointestinal disease					
Parasites					
Respiratory disease					
Bumblefoot					
Absence of toys and objects to interact with					
Living near predator species e.g., dogs and cats					

37. Please add any additional welfare issues not included above that you think commonly affect rat welfare.
Issue 1:
38. Please rate approximately what percentage of the rat population you think are affected by the issue you listed above during their lifetime. {{Q37}}

- 0%
- Less than 10%
- 10-24%
- 25-49%
- 50-74%
- 75% or more
- 100%

39. Please rate the severity of the issue on a scale of 1-5 (1-least severe 5 most severe) i.e. the extent to which you believe the condition would commonly impact upon a single rat's welfare at a given point in time. {{Q37}}

- 1
- 2
- 3
- 4
- 5

40. Please add any additional welfare issues not included above that you think commonly affect rat welfare.
Issue 2:
41. Please rate approximately what percentage of the rat population you think are affected by the issue you listed above during their lifetime. {{Q40}}

- 0%
- Less than 10%
- 10-24%
- 25-49%

- 50-74%
- 75% or more
- 100%

42. Please rate the severity of the issue on a scale of 1-5 (1-least severe 5 most severe) i.e. the extent to which you believe the condition would commonly impact upon a single rat's welfare at a given point in time. {{Q40}}

- 1
- 2
- 3
- 4
- 5

Mouse Welfare:

43. Please rate approximately what percentage of your country's mouse population you think are affected by each of the following issues during their lifetime.

	0%	Less than 10%	10-24%	25-49%	50-74%	75% or more	100%
Small housing e.g., under 80cm x 50cm floor space per 2 mice (4000cm2)							
Lack of mouse companion							
Living with an incompatible mouse which causes fights and/or fear							
Inability to display normal behaviours (e.g., climbing, foraging, gnawing)							
Musculoskeletal disorders							
Tumours/masses							
Inappropriate diet							
Inappropriate handling							
Being underweight							
Being overweight							
Dental issues							
Gastrointestinal disease							
Parasites							

Respiratory disease							
Bumblefoot							
Ocular disease							
Absence of toys and objects to interact with							
Living near predator species e.g., dogs and cats							

44. Please rate the severity of each of the issues on a scale of 1-5 (1-least severe 5-most severe) i.e. the extent to which you believe each condition would commonly impact upon a single mouse's welfare at a given point in time.

	1	2	3	4	5
Small housing e.g., under 80cm x 50cm floor space per 2 mice (4000cm2)					
Lack of mouse companion					
Living with an incompatible mouse which causes fights and/or fear					
Inability to display normal behaviours (e.g., climbing, foraging, gnawing)					
Musculoskeletal disorders					
Tumours/masses					
Inappropriate diet					
Inappropriate handling					
Being underweight					
Being overweight					
Dental issues					
Gastrointestinal disease					
Parasites					

Respiratory disease					
Bumblefoot					
Ocular disease					
Absence of toys and objects to interact with					
Living near predator species e.g., dogs and cats					

45. Please add any additional welfare issues not included above that you think commonly affect mouse welfare.
Issue 1:
46. Please rate approximately what percentage of the mouse population you think are affected by the issue you listed above during their lifetime. {{Q45}}

- 0%
- Less than 10%
- 10-24%
- 25-49%
- 50-74%
- 75% or more
- 100%

47. Please rate the severity of the issue on a scale of 1-5 (1-least severe 5 most severe) i.e. the extent to which you believe the condition would commonly impact upon a single mouse's welfare at a given point in time. {{Q45}}

- 1
- 2
- 3
- 4
- 5

48. Please add any additional welfare issues not included above that you think commonly affect mouse welfare.
Issue 2:
49. Please rate approximately what percentage of the mouse population you think are affected by the issue you listed above during their lifetime. {{Q48}}

- 0%
- Less than 10%
- 10-24%
- 25-49%
- 50-74%
- 75% or more
- 100%

50. Please rate the severity of the issue on a scale of 1-5 (1-least severe 5 most severe) i.e. the extent to which you believe the condition would commonly impact upon a single mouse's welfare at a given point in time. {{Q48}}

- 1

- 2
- 3
- 4
- 5

Chinchilla Welfare:

51. Please rate approximately what percentage of your country's chinchilla population you think are affected by each of the following issues during their lifetime.

	0%	Less than 10%	10-24%	25-49%	50-74%	75% or more	100%
Small housing e.g., under 90cm length x 60cm width per 2/3 chinchillas (5400cm2)							
Lack of chinchilla companion							
Living with an incompatible chinchilla which causes fights and/or fear							
Inability to display normal behaviours (e.g., climbing, foraging, gnawing)							
Musculoskeletal disorders							
Inappropriate diet							
Inappropriate handling							
Being underweight							
Being overweight							
Dental issues							
Gastrointestinal disease							
Parasites							
Respiratory disease							
Bumble foot							
Heatstroke							
Fur slip (releasing chunks of fur as a							

defence mechanism)							
Fur chewing (behavioural problem)							
Ocular disease							
Lack of sand bath							
Absence of toys and objects to interact with							
Living near predator species e.g., dogs and cats							

52. Please rate the severity of each of the issues on a scale of 1-5 (1-least severe 5-most severe) i.e. the extent to which you believe each condition would commonly impact upon a single chinchilla's welfare at a given point in time.

	1	2	3	4	5
Small housing e.g., under 90cm length x 60cm width per 2/3 chinchillas (5400cm2)					
Lack of chinchilla companion					
Living with an incompatible chinchilla which causes fights and/or fear					
Inability to display normal behaviours (e.g., climbing, foraging, gnawing)					
Musculoskeletal disorders					
Inappropriate diet					
Inappropriate handling					
Being underweight					
Being overweight					
Dental issues					

Gastrointestinal disease					
Parasites					
Respiratory disease					
Bumble foot					
Heatstroke					
Fur slip (releasing chunks of fur as a defence mechanism)					
Fur chewing (behavioural problem)					
Ocular disease					
Lack of sand bath					
Absence of toys and objects to interact with					
Living near predator species e.g., dogs and cats					

53. Please add any additional welfare issues not included above that you think commonly affect chinchilla welfare.
Issue 1:
54. Please rate approximately what percentage of the chinchilla population you think are affected by the issue you listed above during their lifetime. {{Q53}}

- 0%
- Less than 10%
- 10-24%
- 25-49%
- 50-74%
- 75% or more
- 100%

55. Please rate the severity of the issue on a scale of 1-5 (1-least severe 5 most severe) i.e. the extent to which you believe the condition would commonly impact upon a single chinchilla's welfare at a given point in time. {{Q53}}

- 1
- 2
- 3
- 4
- 5

56. Please add any additional welfare issues not included above that you think commonly affect chinchilla welfare.
Issue 2:

57. Please rate approximately what percentage of the chinchilla population you think are affected by the issue you listed above during their lifetime. {{Q56}}

- 0%
- Less than 10%
- 10-24%
- 25-49%
- 50-74%
- 75% or more
- 100%

58. Please rate the severity of the issue on a scale of 1-5 (1-least severe 5 most severe) i.e. the extent to which you believe the condition would commonly impact upon a single chinchilla's welfare at a given point in time. {{Q56}}

- 1
- 2
- 3
- 4
- 5

<u>Degu Welfare:</u>

59. Please rate approximately what percentage of your country's degu population you think are affected by each of the following issues during their lifetime.

	0%	Less than 10%	10-24%	25-49%	50-74%	75% or more	100%
Small housing e.g., under 90cm length x 60cm width per 2 degus (5400cm2)							
Lack of degu companion							
Living with an incompatible degu which causes fights and/or fear							
Inability to display normal behaviours (e.g., climbing, foraging, digging, gnawing)							
Musculoskeletal disorders							
Inappropriate diet							
Inappropriate handling							
Being underweight							

Being overweight							
Dental issues							
Gastrointestinal disease							
Parasites							
Respiratory disease							
Bumblefoot							
Vitamin A deficiency							
Diabetes							
Heatstroke							
Tail de-gloving (defence mechanism)							
Lack of sand bath							
Absence of toys and objects to interact with							
Living near predator species e.g., dogs and cats							
Alopecia (due to fur chewing)							
Ocular diseases							

60. Please rate the severity of each of the issues on a scale of 1-5 (1-least severe 5-most severe) i.e. the extent to which you believe each condition would commonly impact upon a single degu's welfare at a given point in time.

	1	2	3	4	5
Small housing e.g., under 90cm length x 60cm width per 2 degus (5400cm2)					
Lack of degu companion					
Living with an incompatible degu which causes fights and/or fear					
Inability to display normal behaviours (e.g., climbing, foraging,					

digging, gnawing)					
Musculoskeletal disorders					
Inappropriate diet					
Inappropriate handling					
Being underweight					
Being overweight					
Dental issues					
Gastrointestinal disease					
Parasites					
Respiratory disease					
Bumblefoot					
Vitamin A deficiency					
Diabetes					
Heatstroke					
Tail de-gloving (defence mechanism)					
Lack of sand bath					
Absence of toys and objects to interact with					
Living near predator species e.g., dogs and cats					
Alopecia (due to fur chewing)					
Ocular diseases					

61. Please add any additional welfare issues not included above that you think commonly affect degu welfare.
Issue 1:
62. Please rate approximately what percentage of the degu population you think are affected by the issue you listed above during their lifetime. {{Q61}}

- 0%
- Less than 10%
- 10-24%
- 25-49%
- 50-74%
- 75% or more
- 100%

63. Please rate the severity of the issue on a scale of 1-5 (1-least severe 5 most severe) i.e. the extent to which you believe the condition would commonly impact upon a single degu's welfare at a given point in time. {{Q61}}

- 1
- 2
- 3
- 4
- 5

64. Please add any additional welfare issues not included above that you think commonly affect degu welfare.
Issue 2:
65. Please rate approximately what percentage of the degu population you think are affected by the issue you listed above during their lifetime. {{Q64}}

- 0%
- Less than 10%
- 10-24%
- 25-49%
- 50-74%
- 75% or more
- 100%

66. Please rate the severity of the issue on a scale of 1-5 (1-least severe 5 most severe) i.e. the extent to which you believe the condition would commonly impact upon a single degu's welfare at a given point in time. {{Q64}}

- 1
- 2
- 3
- 4
- 5

<u>Gerbil Welfare:</u>
67. Please rate approximately what percentage of your country's gerbil population you think are affected by each of the following issues during their lifetime.

	0%	Less than 10%	10-24%	25-49%	50-74%	75% or more	100%
Small housing e.g., under 100cm long, by 40cm wide per 2-4 gerbils (4000cm2)							
Lack of gerbil companion(s)							
Living with an incompatible gerbil which causes fights and/or fear							
Inability to display normal							

behaviours (e.g., climbing, foraging, digging, gnawing)							
Musculoskeletal disorders							
Inappropriate diet							
Inappropriate handling							
Being underweight							
Being overweight							
Dental issues							
Gastrointestinal disease							
Parasites							
Respiratory disease							
Bumblefoot							
Absence of toys and objects to interact with							
Living near predator species e.g., dogs and cats							
Ocular diseases							

68. Please rate the severity of each of the issues on a scale of 1-5 (1-least severe 5-most severe) i.e. the extent to which you believe each condition would commonly impact upon a single gerbil's welfare at a given point in time.

	1	2	3	4	5
Small housing e.g., under 100cm long, by 40cm wide per 2-4 gerbils (4000cm2)					
Lack of gerbil companion(s)					
Living with an incompatible gerbil which causes fights and/or fear					
Inability to display normal					

behaviours (e.g., climbing, foraging, digging, gnawing)					
Musculoskeletal disorders					
Inappropriate diet					
Inappropriate handling					
Being underweight					
Being overweight					
Dental issues					
Gastrointestinal disease					
Parasites					
Respiratory disease					
Bumblefoot					
Absence of toys and objects to interact with					
Living near predator species e.g., dogs and cats					
Ocular diseases					

69. Please add any additional welfare issues not included above that you think commonly affect gerbil welfare.
Issue 1:
70. Please rate approximately what percentage of the gerbil population you think are affected by the issue you listed above during their lifetime. {{Q69}}

- 0%
- Less than 10%
- 10-24%
- 25-49%
- 50-74%
- 75% or more
- 100%

71. Please rate the severity of the issue on a scale of 1-5 (1-least severe 5 most severe) i.e. the extent to which you believe the condition would commonly impact upon a single gerbil's welfare at a given point in time. {{Q69}}

- 1
- 2
- 3

- 4
- 5

72. Please add any additional welfare issues not included above that you think commonly affect gerbil welfare.
Issue 2:
73. Please rate approximately what percentage of the gerbil population you think are affected by the issue you listed above during their lifetime. {{Q72}}

- 0%
- Less than 10%
- 10-24%
- 25-49%
- 50-74%
- 75% or more
- 100%

74. Please rate the severity of the issue on a scale of 1-5 (1-least severe 5 most severe) i.e. the extent to which you believe the condition would commonly impact upon a single gerbil's welfare at a given point in time. {{Q72}}

- 1
- 2
- 3
- 4
- 5

75. If you have any comments about this survey, please enter them here.

Thank you for taking the time to complete this survey. If you know any exotic specialists, please share the survey link with them.
